# MR-guided focused ultrasound increases antibody delivery to nonenhancing high-grade glioma

**DOI:** 10.1093/noajnl/vdaa030

**Published:** 2020-03-05

**Authors:** Caterina Brighi, Lee Reid, Alison L White, Laura A Genovesi, Marija Kojic, Amanda Millar, Zara Bruce, Bryan W Day, Stephen Rose, Andrew K Whittaker, Simon Puttick

**Affiliations:** 1 Australian Institute for Bioengineering and Nanotechnology, The University of Queensland, Brisbane, Australia; 2 ARC Centre of Excellence in Convergent Bio-Nano Science and Technology, The University of Queensland, Brisbane, Australia; 3 Commonwealth Scientific and Industrial Research Organization, Australian e-Health Research Centre, Royal Brisbane and Women’s Hospital, Brisbane, Australia; 4 Department of Cell and Molecular Biology, QIMR Berghofer Medical Research Institute, Brisbane, Australia; 5 School of Biomedical Sciences, Faculty of Health, Queensland University of Technology, Brisbane, Australia; 6 School of Biomedical Sciences, The University of Queensland, Brisbane, Australia; 7 Institute for Molecular Bioscience, The University of Queensland, Brisbane, Australia

**Keywords:** blood, brain barrier, focused ultrasound, high-grade glioma, MRI, vasculature permeability

## Abstract

**Background:**

High-grade glioma (HGG) remains a recalcitrant clinical problem despite many decades of research. A major challenge in improving prognosis is the inability of current therapeutic strategies to address a clinically significant burden of infiltrating tumor cells that extend beyond the margins of the primary tumor mass. Such cells cannot be surgically excised nor efficiently targeted by radiation therapy. Therapeutic targeting of this tumor cell population is significantly hampered by the presence of an intact blood–brain barrier (BBB). In this study, we performed a preclinical investigation of the efficiency of MR-guided Focused Ultrasound (FUS) to temporarily disrupt the BBB to allow selective delivery of a tumor-targeting antibody to infiltrating tumor.

**Methods:**

Structural MRI, dynamic-contrast enhancement MRI, and histology were used to fully characterize the MR-enhancing properties of a patient-derived xenograft (PDX) orthotopic mouse model of HGG and to develop a reproducible, robust model of nonenhancing HGG. PET–CT imaging techniques were then used to evaluate the efficacy of FUS to increase ^89^Zr-radiolabeled antibody concentration in nonenhancing HGG regions and adjacent non-targeted tumor tissue.

**Results:**

The PDX mouse model of HGG has a significant tumor burden lying behind an intact BBB. Increased antibody uptake in nonenhancing tumor regions is directly proportional to the FUS-targeted volume. FUS locally increased antibody uptake in FUS-targeted regions of the tumor with an intact BBB, while leaving untargeted regions unaffected.

**Conclusions:**

FUS exposure successfully allowed temporary BBB disruption, localized to specifically targeted, nonenhancing, infiltrating tumor regions and delivery of a systemically administered antibody was significantly increased.

**Key Points**

- Our patient-derived xenograft mouse model of HGG reproduces infiltrating tumor with an intact BBB.
- FUS disrupts the BBB and selectively increases antibody uptake in targeted nonenhancing HGG.
- FUS treatment is localized and leaves untargeted regions unaffected.

**Importance of the Study**

The ability to target a tumor burden lying behind an intact BBB is paramount to improving the treatment of HGG patients. FUS shows significant clinical potential, allowing for both selective targeting and efficient delivery of systemic therapies to a localized area of the tumor. To the best of our knowledge, there have been no preclinical studies that demonstrate the efficacy of FUS in animal models of intracranial HGG with an intact BBB, leading to a lack of evidence that FUS will improve uptake of systemic therapies in infiltrative regions of HGG. Here we demonstrate, for the first time, that FUS can selectively open the BBB to enhance the delivery of a targeted antibody in a PDX mouse model that reproducibly forms infiltrating HGG with an intact BBB. Overall, our results highlight the potential of FUS to change the clinical management of HGG, creating new therapeutic possibilities for emerging systemic therapies.

One of the biggest challenges in the delivery of systemic therapy for high-grade glioma (HGG) is the heterogeneous vasculature morphology that characterizes the tumor. HGG exhibits a highly heterogeneous mix of neovascular mechanisms and environments, often with angiogenesis, vascular co-option,^1^ vascular mimicry,^2^ and even glioblastoma-endothelial cell transdifferentiation^3^ appearing within the same tumor.^4,5^ This leads to a high degree of heterogeneity in the status of the blood–brain barrier (BBB) across the tumor,^6^ including regions of invasive tumor in the majority of HGG where the BBB is fully intact.^7^ These regions of tumor tissue present a significant barrier to the delivery of systemic therapies and must be addressed if the patient prognosis is to be improved.^7^

Standard clinical treatment for HGG involves surgical resection followed by chemotherapy and radiotherapy where both resection margins and external beam radiotherapy margins are planned based on a contrast-enhanced (CE) MRI.^8,9^ Delivery of contrast agents to tumorous tissue relies on the disruption of the BBB, thus there is often residual disease following therapy ultimately leading to patient relapse.^7,10,11^ As such, these residual nonenhancing regions of HGG are an important target for emerging therapies.

A number of neurosurgical techniques that temporarily disrupt the BBB and allow for more effective drug delivery have been investigated to overcome this challenge.^12^ Of these, MR-guided Focused Ultrasound (FUS) holds particular promise and is beginning to have an impact in the clinical domain. FUS involves the application of a focused beam of low-frequency ultrasonic waves to a specific region of the tumor tissue guided by an MRI. When used in combination with systemic delivery of echogenic microbubbles, FUS produces a transient disruption of the BBB that lasts up to 24 h^13^ (Figure 1). This strategy holds significant promise for HGG, allowing a high payload of systemic therapy to be delivered to a localized area of the tumor without affecting the surrounding healthy brain tissue.^14^

**Figure 1.**
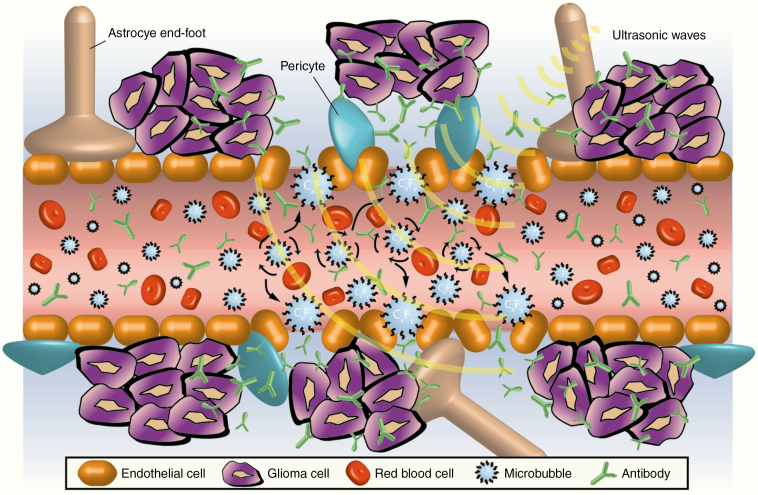
Mechanism of Focused Ultrasound (FUS)-induced BBB disruption. Application of a FUS pulse at the resonance frequency of systemically administered microbubbles causes stable cavitation. This leads to temporary disruption of endothelial tight junctions allowing systemically administered antibodies to penetrate into the brain parenchyma and reach glioma cells.

There are now a number of preclinical^13,15–19^ and clinical^20^ examples and 4 ongoing clinical trials (NCT03712293, NCT02343991, NCT03322813, and NCT03616860; clinicaltrials.gov), evaluating the safety and effectiveness of using FUS in combination with standard systemic therapies in brain tumors. A class of systemic therapy that has shown limited success in brain cancer, despite significant efficacy in non-CNS cancers, is monoclonal antibodies. A dominant factor in the limited success of monoclonal antibody therapies in HGG is the inability of such large macromolecules to extravasate in the presence of a functional BBB.^21–24^ FUS represents a very promising strategy to overcome this limitation.^25^ Several studies have reported positive results when using FUS to enhance antibody penetration across the BBB for the treatment of Alzheimer’s disease^25–28^and a small number of studies have investigated the effects of combining FUS with antibody delivery on tumor growth, immune response, and animal survival in mouse or rat models of glioma.^29–33^ While these studies reported promising preclinical results, there are a number of critical limitations that must be addressed before the clinical translation is considered.

The limited number of preclinical investigations reported to date has been carried out either in mouse models of breast cancer brain metastases, in the C6 rat model of glioma, or in the U87 mouse model of glioma, all of which show significant volumes of contrast enhancement prior to treatment with FUS.^30–33^ This indicates that the BBB is already compromised in these models and so are not faithful representations of the true disease state. While all studies showed an increase in the concentration of the molecule of interest in the tumor tissue following FUS, there is limited evidence that these strategies would be effective in treating nonenhancing HGG. It is often suggested that FUS-induced extravasation of molecules of interest in the healthy brain is sufficient evidence to infer that FUS will increase extravasation in nonenhancing HGG tissue.^16,25,29,33–36^ However, due to the significant differences in vascular architecture and regional blood flow between a healthy brain and nonenhancing HGG tissue, it is unlikely that mechanisms of FUS-induced BBB disruption will be consistent between a healthy brain and nonenhancing HGG tissue.

In this work, we fully characterize the MR-enhancing properties of a patient-derived xenograft (PDX) orthotopic mouse model of HGG to develop a robust and reproducible model of nonenhancing HGG. Using this model, we demonstrate the efficacy of FUS to increase ^89^Zr-radiolabelled antibody concentration in nonenhancing HGG tissue.

## Methods

### Experimental Design

All animal experiments were approved by both the University of Queensland Animal Ethics Committee and the QIMR Berghofer Animal Ethics Committee. Experimental animal care guidelines were adhered to at all times. Fourteen NOD/SCID tumor-bearing mice were used for this study. Animals were randomly divided into 2 groups: a control group of 6 mice and a FUS-treated group of 8 mice. Before each procedure and imaging session, the mice were anesthetized by inhalation of 2% isoflurane (Isothesia NXT; Henry Schein Animal Health) in the air (2 L/min).

Structural MRI was used to assess tumor size and extent of BBB opening. T_2_-weighted images (T2) were acquired to characterize the entire tumor size, including regions of edema. Contrast-enhanced T1-weighted MRI images (T1-CE) were used to determine the extent of BBB opening in the tumor prior to and after FUS treatment. Dynamic contrast-enhanced T1-weighted MRI imaging (DCE) was used to characterize the permeability of the BBB in different areas of the tumor.

Tumor development was monitored for 4 months via T2 imaging. Once the tumor reached a size of 200 ± 100 mm^3^ the mice were enrolled in the experiment (Supplementary Figure S1). On Day 1 the fur on the scalp of the FUS group of mice was completely removed with clippers followed by application of depilatory cream (Veet), then a T2, T1 maps, a DCE sequence, and a T1-CE were acquired for both groups. DCE sequences were acquired only for 12 of the 14 mice. On Day 2, both groups were administered a dose (~4 MBq) of ^89^Zr-radiolabelled antibody targeting EphA2 receptors. Following antibody administration, the FUS group was subjected to FUS treatment. A T1-CE was acquired immediately following FUS exposure to observe the extent of FUS-induced BBB opening. To assess the accumulation of the radiolabeled antibody in tumor tissue in both the control and the FUS groups a PET–CT scan was acquired on Day 3 at 24 h post-antibody injection or post-FUS treatment, respectively. All mice were euthanized by cardiac perfusion with ice-cold phosphate-buffered saline (PBS) followed by 4% paraformaldehyde (PFA) 72 h post-injection of the radiolabeled antibody (Day 5). The brains were excised and stored in 4% PFA at 4°C, washed twice with 0.1% NaN_3_ in PBS after 24 and 48 h, and then stored in 70% ethanol at 4°C until embedding in paraffin. Finally, histology was performed on the paraffin-embedded brains.

### Tumor Model

The HGG tumor model used in this study was generated by orthotopic injection of WK1 neurospheres into the right striatum of 6-week-old female NOD/SCID mice. The WK1 cell line was derived from a 77-year-old man with right parieto-occipital glioblastoma prior to him receiving chemotherapy or radiotherapy. Tumor tissue was collected as part of a study approved by the Human Ethics Committees of the QIMR Berghofer Medical Research Institute and the Royal Brisbane and Women’s Hospital with full patient consent. Full cell line characterization data are publicly available from Q-Cell https://www.qimrberghofer.edu.au/q-cell/.^37^ Cell culture and xenograft model implant methodology, together with studies on median survival of NOD/SCID mice with implanted WK1 xenografts, were previously published.^38^

### Antibody

The EphA2-4B3 antibody used in this work is an IgG2a antibody raised in wild-type mice against a human EphA2-Fc immunogen. Antibody production using a standard hybridoma and subsequent purification was carried out at the Protein Expression Facility at The University of Queensland. Studies reporting the characterization of EphA2 receptor expression in WK1 xenografts and data on the specificity and affinity of the 4B3 antibody to the EphA2 receptor have been previously published by our group.^38,39^

### Magnetic Resonance Imaging

MR images were acquired on a Bruker 7T Clinscan interfaced with a Siemens spectrometer running Numaris 4 VB17 using a 23 mm mouse head volume coil. A catheter preloaded with the gadolinium contrast agent (CA) (gadobutrol, 0.1 mmol/kg, Gadovist 1.0; Bayer) was placed in the tail vein. Imaging sequences included a T2 (resolution 0.078 × 0.078 × 0.700 mm^3^; TR/TE 2750/45 ms/ms; flip angle 180°), T1 maps (resolution 0.195 × 0.195 × 0.850 mm^3^; TR/TE 12/0.93 ms/ms; flip angles 10°, 15°, 20°, 25°, 30°), DCE (resolution 0.195 × 0.195 × 0.850 mm^3^; TR/TE 12/0.93 ms/ms; flip angle 12°), and T1-CE (resolution 0.117 × 0.117 × 0.120 mm^3^; TR/TE 12/1.78 ms/ms; flip angle 21°). DCE images were acquired before, during, and after injection of the gadolinium bolus (40 μL at a rate of 10 μL/s).

### FUS Sonication

FUS was performed using an LP-100 FUS instrument (FUS Instruments) using a 1.1 MHz hemispherical transducer mounted in a 3-axis positioning system aligned with the MRI coordinate space. Mice were anesthetized and laid in the supine position on the sonication system with the dorsal surface of the head centered over the FUS transducer. Breathing was visually monitored throughout the experiment and mice were kept warm with a heat lamp positioned over the bed (Supplementary Figure S2B). Prior to sonication, the FUS bed with the mouse secured in position was moved into the MR scanner and a T2 image was acquired to visualize the tumor mass. The image was imported into the FUS guidance software and a sonication volume consisting of 10–20 target points was defined across the tumor (Supplementary Figure S2A). The FUS bed with mouse secured in position was transferred to the FUS system and the mouse was injected with radiolabeled antibody solution prior to sonication. Sonication consisted of 10 ms focused ultrasonic bursts delivered transcranially to the target points over a period of 2500 ms with a total sonication time of 120 s. The acoustic power level used corresponded to a peak rarefactional focal pressure amplitude in water of 0.85 MPa. During sonication a combined solution of gadolinium CA (1:10 dilution) and activated ultrasound CA microbubbles diluted to 2% in MilliQ water (Definity; Lantheus Medical Imaging) was administered intravenously with a catheter placed in the tail vein as a 200 μL infusion over 60 s. The Definity microbubbles were activated 5 min prior to sonication by vigorous shaking with a VialMix (Lantheus Medical Imaging) for a pre-set time of 45 s. Immediately following microbubble activation, the suspension contained approximately 1.2 × 10^10^ microbubbles/mL with a mean diameter range of 1.5–2.9 μm as measured by Beckman Coulter Counter Multisizer (Supplementary Figure S3).

### PET–CT Imaging

^89^Zr radiolabeling was performed as described in the work of Zeglis and Lewis^40^ and radiochemical yield and purity were determined by thin layer chromatography (TLC). Doses were administered if the radiochemical purity was more than 95%. PET–CT images were acquired 24 h post-FUS using an Inveon Preclinical PET-CT system (Siemens). Mice were anesthetized and maintained using 2% isoflurane in oxygen at a flow rate of 2 L/min and positioned in an in-house manufactured 4-mouse scanning bed. A 30 min PET image was acquired followed by a CT for attenuation correction and co-registration to the MRI data. The PET images were reconstructed using the OSEM-2D reconstruction algorithm in the Inveon Acquisition Workspace (IAW, Siemens) correcting for attenuation and ^89^Zr detector efficiency.

### Histology and Microscopy

Paraffin-embedded mouse brains were cut into 7-μm-thick coronal sections using a Rotary Microtome HM 355 S (Microm International). Hematoxylin and eosin (H&E) staining was used to assess tumor margins. Following deparaffinization, slides were stained in hematoxylin (Sigma Aldrich) for 3 min and the excess of hematoxylin was removed by short immersion of slides in 1% HCl, followed by 0.1% LiCO_**3**_. Next, slides were stained in Eosin Y solution (Sigma Aldrich) for 30 s and dehydrated by using 70%, 90%, and 100% ethanol for 30 s each, followed by xylene for 10 min. Slides were mounted with Entellan mounting medium (ProSciTech) and dried for 2 h. Expression of glial fibrillary acidic protein (GFAP) and ionized calcium-binding adaptor molecule 1 (Iba1) was assessed by immunofluorescence staining to interrogate changes in astrocyte and microglia activation in the tumor vasculature. The staining was performed using a standard protocol, including heat-activated, citrate-based pH 6.0 antigen retrieval and blocking with MOM kit (BMK-2202; Vector Laboratories). The primary antibodies used were mouse anti-GFAP (MAB360; 1:100 dilution; Merck) and rabbit anti-Iba1 (019-19741; 1:400 dilution; FUJIFILM Wako Chemicals USA Corp). Secondary antibodies used were donkey anti-mouse A488 (ab150105; 1:250 dilution; Abcam) and donkey anti-rabbit A594 (A21207; 1:250 dilution; ThermoFisher Scientific). Primary and secondary antibodies were diluted in MOM kit diluent (BMK-2202; Vector Laboratories). Images were captured using the Aperio Brightfield XT slide scanner (ScanScope XT) and Axiovert 200 inverted confocal microscope with LSM 710 scanner (Carl Zeiss Pty Ltd) as Z-stacks and presented as the sum of the Z-stack projection. Whole-brain images were acquired as tiled image stacks. Image processing was performed by using ImageScope and ImageJ softwares.

### Image Analysis

#### Structural MRI and PET image analysis

**Figure 2.**
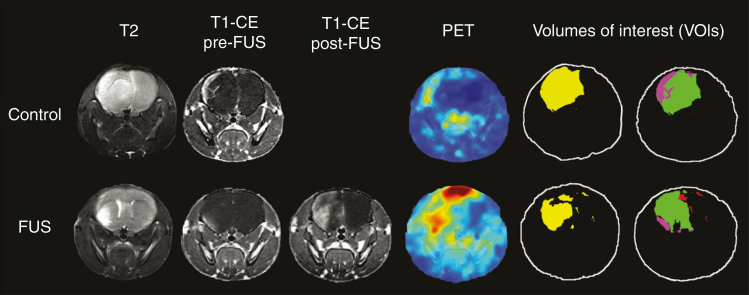
Example of VOI segmentation in different imaging modalities. (L–R) T2, T1-CE pre-FUS, T1-CE post-FUS, and PET images of a mouse from the control and FUS groups followed by segmented tumor VOI masks (yellow), masks of CE tumor VOI (purple), targeted non-CE tumor VOI (green), and non-CE tumor post-FUS VOI (red). Note that for the control mouse the non-CE tumor post-FUS VOI is not highlighted as it corresponds to the targeted non-CE tumor VOI (green). Overlays of the masks on the MRI images from which they were generated are illustrated in Supplementary Figure S4.

*—*DICOM images were converted into NIFTI format using a combination of dcm2niix and MRtrix3.^41,42^ The radioactivity concentrations in the PET images were decay corrected using a ^89^Zr half-life of 78.41 h using in-house software. T2, CT, and decay-corrected PET images were then rigidly registered to the post-contrast T1-CE using ANTS^43^ and linearly resampled into this space. Binary masks of the volumes of interest (VOIs) were manually delineated for both the T2 and T1-CE images using a semiautomatic active contour segmentation tool (ITK-SNAP^44^; Figure 2 and Supplementary Figure S4). These included the tumor, CE tumor, non-CE tumor, FUS-treated non-CE tumor, targeted non-CE tumor, and non-CE tumor post-FUS VOIs. Masks of tumor VOI were defined from hyperintense regions on the T2 images, masks of CE tumor VOIs for both groups and targeted non-CE tumor VOIs for the control group were defined on the T1-CE pre-FUS images, masks of targeted non-CE tumor VOIs and non-CE tumor post-FUS VOIs for the FUS group were defined on the T1-CE post-FUS images. Name, origin, and description of the VOIs are provided in Table 1.

**Table 1.** Name, Origin, and Description of VOIs

Name of VOI	Image of Origin for VOI	Description of VOI
Tumor	T2	Entire tumor area, including regions of edema
CE tumor	T1-CE pre-FUS	Originally CE tumor area
Non-CE tumor	Subtraction of CE tumor VOI from tumor VOI	Originally non-CE tumor area
FUS-treated non-CE tumor	Subtraction of CE tumor VOI pre-FUS from CE tumor VOI post-FUS	Region of the non-CE tumor with an open BBB as a result of the FUS treatment
Targeted non-CE tumor (control)	Non-CE tumor VOI	Originally non-CE tumor area in control mice
Targeted non-CE tumor (FUS)	FUS-treated non-CE tumor VOI	Non-CE tumor regions targeted by FUS
Non-CE tumor post-FUS	Subtraction of CE tumor VOI post-FUS from tumor VOI	Region of the non-CE tumor not targeted by FUS

Statistics for each VOI, including volume in voxels and mm^3^ and mean intensity values, were calculated using the fslstats (FSL^45^) neuroimaging analytical tool. The volumetric ratio of the CE tumor VOI to the tumor VOI represents the proportion of the entire tumor with a disrupted BBB. This ratio was used to calculate and compare the extent of BBB disruption in the tumor prior to and after FUS treatment. The volumetric change of the non-CE tumor was used as a measure of the FUS-induced BBB opening. The dose of radiolabeled antibody in the targeted non-CE tumor, CE tumor, and non-CE tumor post-FUS was obtained by masking the PET images by the relevant binary VOIs (Figure 2 and Supplementary Figure S4). Specifically, the targeted non-CE tumor was represented by the non-CE tumor VOI for the control group and by the FUS-treated non-CE tumor VOI for the FUS group. Mean values of radiolabeled antibody uptake in the VOIs were calculated as a percentage of injected dose per gram of brain (% ID/g) in each mouse and these values were used to calculate the average of mean dose uptake in each of the 2 experimental groups. It was assumed that 1 g of brain is equal to 1 cm^3^ in these calculations.

#### DCE sequence image analysis

—T1 maps and DCE sequences were imported into Nordic-ICE (NordicNeuroLab) and used to extract curves of change in 1/*R*_1_ signal enhancement in relevant 2D regions of interest (ROIs). Image preprocessing consisted of noise correction, motion artifact rectification, T1 maps baseline correction, and signal normalization by a selection of population-based arterial input functions, obtained from the average of the arterial input functions of 25 previously scanned WK1 mice. Curves of change in 1/*R*_1_ signal enhancement were calculated in ROIs of the size of approximately 1 mm^2^ in the CE tumor VOI and in the non-CE tumor VOI.

#### Fluorescence imaging quantification analysis

—For the quantitation of GFAP and Iba1 staining, images were processed for contrast, brightness, and color in Adobe Photoshop software. Three fields of view were analyzed for each brain section, and a total of 3 FUS-treated and 3 control animals were included in the study. The integrated GFAP and Iba1 staining was quantified for each field of view separately by measuring mean pixel intensity in ImageJ. For each image, background intensity was subtracted from the mean pixel intensity.

### Statistical Analysis

Statistical analyses were performed using GraphPad Prism 7 Software. The two-tailed, paired nonparametric Wilcoxon matched-pairs signed-rank test, *α* = 0.05, was used to determine significance in the comparison of the extent of BBB opening before and after FUS treatment for the FUS group. Two-tailed *t*-tests with Welch’s correction, *α* = 0.05, were applied to the comparison of pixels mean fluorescence intensity between the control and the FUS groups in the GFAP and Iba1 quantification analyses. Two-tailed, unpaired Mann–Whitney *U*-tests, *α* = 0.05, were applied to the comparison of mean antibody uptake in the targeted non-CE tumor, CE tumor, and non-CE tumor post-FUS between the control and the FUS groups. Two-tailed Pearson correlation, *α* = 0.05, and linear regression analysis were used to assess the linear correlation between antibody uptake in the FUS-treated non-CE tumor VOIs and the extent of FUS-induced BBB opening.

## Results and Discussion

### Characterization of the BBB in the WK1 Mouse Model

**Figure 3.**
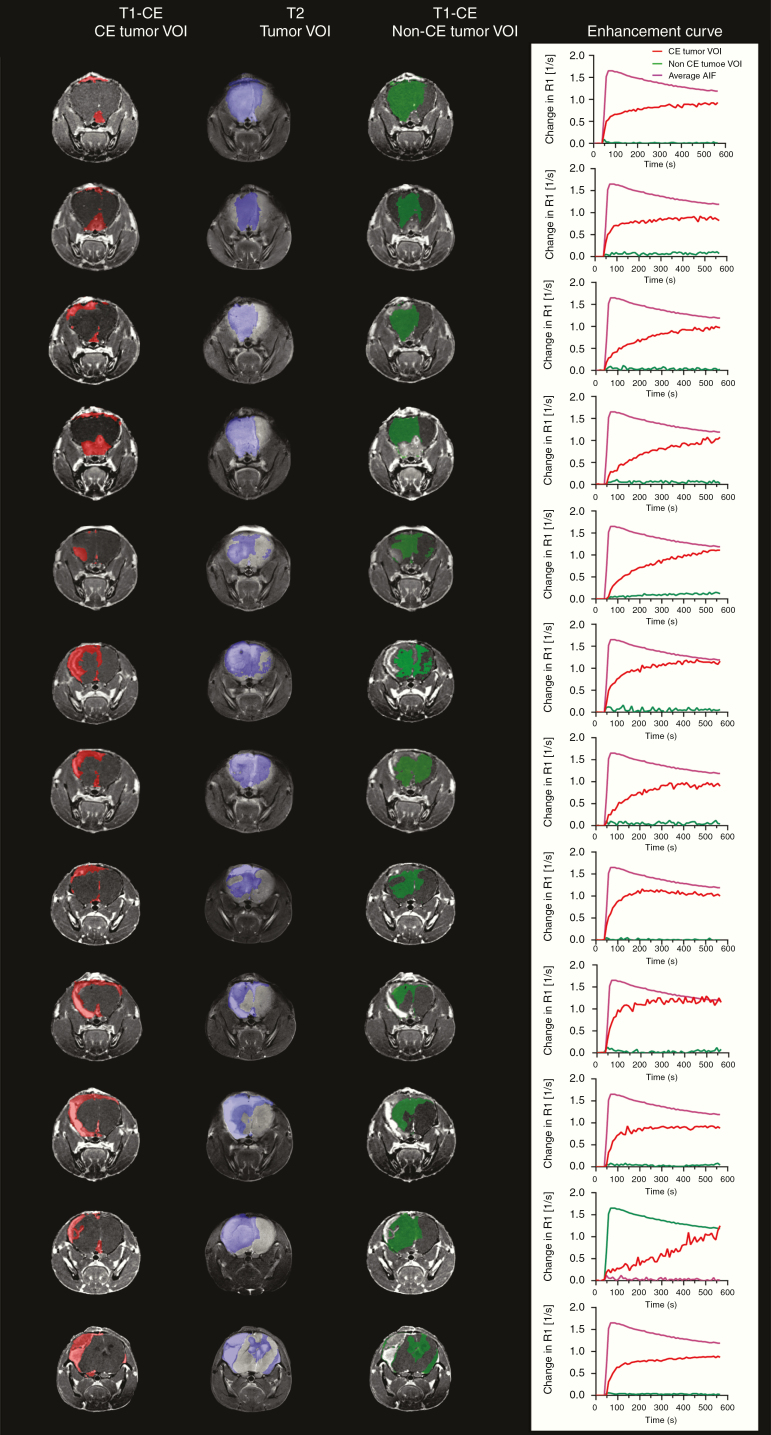
Tumor characteristics and T1 signal relaxation enhancement curves in the non-CE tumor VOI for 12 WK1 mice that underwent a DCE MRI scan. (L–R) T1-CE with overlaid CE tumor VOI (red), T2 with overlayed tumor VOI (blue), T1-CE with overlayed non-CE tumor VOI (green), and T_1_ signal relaxation enhancement curves from within the non-CE tumor VOI.

DCE imaging was used to characterize the permeability of the BBB in different areas of the tumor of the WK1 mice. As extensively described in the literature, the leakage of the CA across the BBB can be assessed by measuring the change in T1 signal (1/*R*_1_) of the tissue over time.^46^ In our study, we qualitatively compared the T1 signal enhancement curves in a 1 mm^2^ ROI within the CE tumor VOI and a 1 mm^2^ ROI within the non-CE tumor VOI for 12 WK1 mice. As shown in Figure 3, the T1 enhancement curves in the CE tumor ROIs show an increase in signal enhancement following bolus injection and retention of this signal enhancement over time. This behavior indicates extravasation of the CA from the leaky vasculature into the brain and retention, which is strong evidence for a disrupted BBB. On the other hand, the T1 enhancement curves in the non-CE tumor ROIs show negligible signal enhancement, which reflects the absence of CA leakage into the brain and, consequently, an intact BBB in this area of the tumor. These results highlight the similarity of the vasculature characteristics of this tumor model with the majority of HGG patients, who present both regions of the angiogenic tumor with a disrupted BBB and regions of infiltrating tumor with an intact BBB.^7^ This characteristic is particularly important for a preclinical study aiming at assessing the efficiency of FUS to enhance drug delivery and alleviate the infiltrating tumor burden, as it provides a means to selectively target nonenhancing infiltrating tumor regions.

### Characterization of FUS-Induced BBB Opening

**Figure 4.**
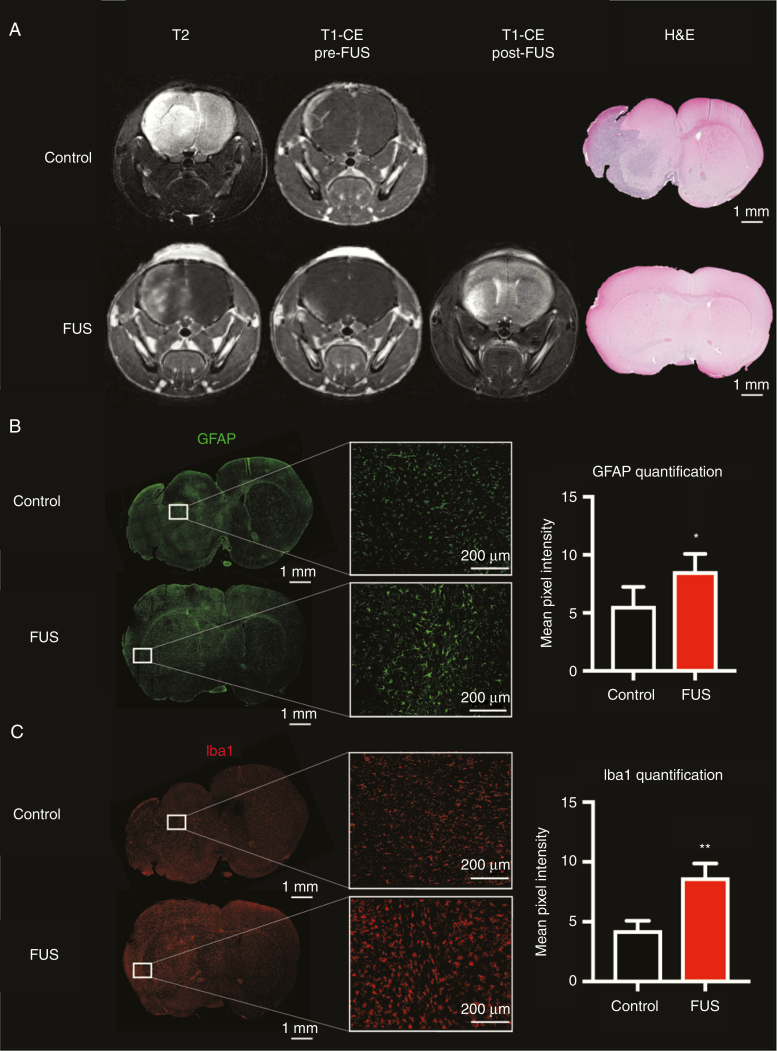
Example of tumor characteristics and FUS treatment effects of a control mouse and a FUS mouse. (A) T2 images, T1-CE pre-FUS, T1-CE post-FUS images, and H&E-stained brain sections; (B) GFAP-stained sections and GFAP quantification analysis; (C) Iba1-stained sections and Iba1 quantification analysis. Higher-magnification (20×) images of GFAP and Iba1 stained sections are taken in regions of non-CE tumor in the control mouse and in regions of FUS-treated tumor in the FUS mouse.

Structural MRI was used in this study to assess tumor size and extent of BBB opening, as described in the Methods section. The area of hyperintensity in the T2 was found to be larger than the area of contrast enhancement in the T1-CE pre-FUS for all mice (Figure 4A and Supplementary Figure S5A). This confirms that the region of a tumor with an originally disrupted BBB represented only a small area of the tumor in this model. T1-CE images acquired before and after FUS showed an increase in contrast-enhancing area, indicating that the FUS treatment successfully induced further BBB opening. This is a particularly important and novel aspect of our study, uniquely demonstrating that FUS can selectively alter the vasculature in the non-CE tumor.

Histological analysis of the brain sections of FUS-treated and control mice was performed in order to characterize ex vivo the tumor margin and capacity of tumor infiltration. The H&E staining of the brain sections shown in Figure 4A and Supplementary Figure S5A demonstrated that the tumor tissue boundaries generally corresponded to the boundaries of the hyperintense regions on the T2, indicating that our in vivo method to characterize the entire tumor volume was appropriate. Moreover, the staining revealed that infiltrating tumor regions usually corresponded to regions of the tumor that had an intact BBB (non-CE on the T1-CE). In contrast, meningeal non-infiltrating tumor regions, which are highly angiogenic, usually corresponded to CE tumor regions.

Disruption of the BBB induces activation of astrocytes and microglia as a result of a sterile inflammatory response.^47–50^ In order to screen for potential gliosis induced by loss of vascular integrity caused by FUS treatment, we used GFAP and Iba1 as markers of activated astrocytes and microglia, respectively. A moderate and nonuniform astrocyte activity was observed in the tumors of control mice on the basis of GFAP expression, while severe astrogliosis was found in the FUS mice in regions of the tumor targeted with FUS (*P* = .0394, two-tailed *t*-test with Welch’s correction; Figure 4B and Supplementary Figure S5B). This suggests that FUS treatment induces an inflammatory response concomitant with the tumor vascular disruption and is in agreement with previously reported data.^49,50^ To further confirm an inflammation response, we screened for Iba1 expression and showed a significant microgliosis in the FUS-treated tumors relative to untreated controls (*P* = .0083, two-tailed *t*-test with Welch’s correction; Figure 4C and Supplementary Figure S5C). Once activated, microglia further impair BBB function by modulating the expression of tight junctions, which are essential for the BBB integrity and function.^51^ Despite the fact that no sign of tumor tissue or vascular damage was found in the H&E-stained sections of the FUS mice brains, the immunofluorescence revealed substantial astrogliosis and microgliosis caused by the FUS-induced vascular disruption in the brain sections harvested 3 days post-treatment. This is in line with the previous findings suggesting that astrocytes and microglia activation can be detected as long as 2 weeks post-FUS.^52^

### Quantification of the Extent of FUS-Induced BBB Opening and Correlation With Antibody Tumor Uptake

**Figure 5.**
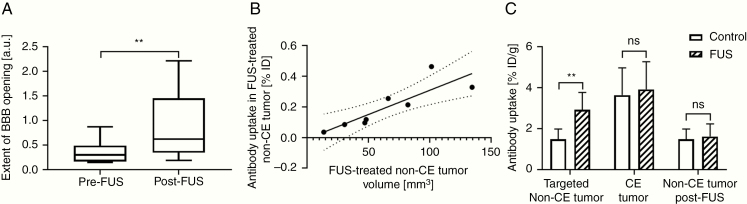
Quantitative analysis of FUS-induced degree of BBB opening and values of antibody tumor uptake in different VOIs. (A) The plot shows the median extent of BBB opening in the tumor of FUS mice prior and post-FUS treatment. (B) The plot shows the correlation between the percentage of antibody tumor uptake in the FUS-treated non-CE tumor and the total volume of the FUS-treated non-CE tumor. (C) The chart shows the comparison of mean values of antibody uptake in CE tumor, targeted non-CE tumor, and non-CE tumor post-FUS between the FUS group and the control group.

The volumetric change of the non-CE tumor was used as a measure of the extent of FUS-induced BBB opening. As shown in Figure 5A, the median extent of BBB opening was significantly higher post-FUS than pre-FUS treatment (*P* = .0078, Wilcoxon matched-pairs signed ranked test), indicating that FUS treatment temporarily increased BBB permeability.

In order to determine whether the extent of FUS-induced BBB opening corresponds to a proportionally higher uptake of tumor-targeting antibody, we examined the relationship between antibody uptake in the FUS-treated non-CE tumor VOIs and the volume of FUS-treated non-CE tumor VOIs (Figure 5B). A statistically significant (Pearson *r* coefficient 0.8621, *P* = .0059) direct linear correlation was found, which implies that the amount of antibody uptake in tumor with an intact BBB can be proportionally increased by increasing the efficacy of the FUS treatment. While often overlooked, this linear relationship is extremely important when assessing the potential of FUS to increase drugs’ uptake. This is because T1-CE imaging provides a means to quantify the extent of tumor volume with a disrupted BBB by passage only of the gadolinium CA (~605 Da); it does not provide meaningful predictions about the ability for larger molecules, such as antibodies in the range of approximately 150 kDa, to cross the disrupted BBB. Furthermore, although the precise molecular mechanisms by which antibodies penetrate into the tumor upon FUS are still unknown, there is evidence that FUS-induced antibody uptake into the tumor is not necessarily only driven by passive diffusion^53^ and can therefore differ from the uptake mechanism seen for smaller molecules. Thus, it is important to establish the relationship between the uptake of the specific antibody used and the extent of FUS-induced BBB opening in every preclinical study evaluating the ability of FUS to enhance antibody penetration into the tumor.

### FUS Increases Antibody Uptake Only in Targeted Non-CE Tumor

In order to determine the effects of FUS treatment on antibody uptake in different regions of the tumor, changes in antibody uptakes were compared in both the CE and non-CE tumor regions with and without treatment. In the non-CE tumor regions targeted with FUS, antibody uptake increased significantly more in FUS-treated mice than in control mice (*P* = .0013, Mann–Whitney *U*-test; Figure 5C). This shows, for the first time, that FUS can temporarily open the BBB in invasive brain tumor tissue to increase uptake of antibody-targeted therapies in regions of the tumor that would otherwise be inaccessible to macromolecular drugs.

Further examination revealed that the FUS treatment did not significantly increase (*P* = .7546, Mann–Whitney *U*-test) the mean antibody uptake in the regions that were contrast enhancing prior to FUS (Figure 5C). This result indicates that FUS did not significantly improve the transport of antibody drugs across an already disrupted BBB.

Finally, the effect of FUS treatment on antibody uptake in regions of non-CE tumor adjacent to the targeted non-CE tumor was analyzed, by comparing the mean values of antibody uptake in the non-CE tumor post-FUS VOIs between the 2 groups. Figure 5C illustrates that there was no statistically significant difference between the 2 groups (*P* = .8518, Mann–Whitney *U*-test), indicating that the FUS treatment was highly localized and did not affect interstitial transport of antibodies beyond the targeted volume.

## Conclusions

One of the greatest challenges associated with the treatment of HGG is the delivery of systemic therapy to a clinically significant infiltrative tumor that is protected by an intact BBB. In this study, we investigated the effect of using FUS to temporarily disrupt the BBB in regions of the tumor with an intact BBB to facilitate increased uptake of a targeted antibody.

Using serial in vivo MRI techniques in combination with ex vivo histology, we show that the WK1 primary HGG mouse model has significant tumor burden and intact BBB.

Using this mouse model, we developed an approach to quantify the extent of FUS-induced BBB opening in regions of the tumor with an intact BBB and that by increasing the volume of FUS-induced CE tumor, we can proportionally increase the amount of antibody uptake in these regions.

We show, for the first time, that FUS can locally increase antibody uptake in FUS-targeted regions of an HGG animal model with an intact BBB, while leaving untargeted regions unaffected. Interestingly, we observed no significant effect of FUS in regions of tumor that were contrast enhancing prior to treatment with FUS. These results indicate that observations made in models where the BBB is fully disrupted prior to the application of FUS should be carefully considered.

It is clear that FUS has significant potential to increase the efficacy of systemic therapies in HGG; however, we believe it is also clear that the choice of preclinical model and careful experimental design are paramount to furthering the understanding of this potential. We believe that our results are a pioneering demonstration of the potential of FUS to improve therapeutic delivery in non-CE brain tumors and that this paradigm should be explored in the clinical domain.

## Funding

This work was supported by the Australia Research Council Centre of Excellence in Bio-Nano Science & Technology (CE140100036); Australia Research Council Discovery Program (DP110104299, DP180101221); Commonwealth Scientific and Industrial Research Organization Probing Biosystems Future Science Platform; the Cure Brain Cancer Foundation (R14/2173 to S.P. and 022872 to L.A.G.); the Children’s Hospital Foundation (50214 to C.B. and 023744 to L.A.G.); Advance Queensland Research Fellowship (R-09964-01 to L.R.). In addition, the authors acknowledge the facilities and scientific and technical assistance of the National Imaging Facility, a National Collaborative Research and Infrastructure Strategy (NCRIS) capability, at the Centre for Advanced Imaging, University of Queensland.

*Conflict of interest statement*. The authors declare no competing financial interests.

## Authorship Statement.

Research design: C.B., S.P., A.K.W., and S.R. Experiments performance: C.B., S.P., Z.B., L.A.G., A.M., M.K., and A.L.W. Data analysis: C.B., L.R., and M.K. Data interpretation: C.B., S.P., A.L.W., S.R., A.K.W., M.K., and L.A.G. Manuscript preparation: C.B., L.R., L.A.G., M.K., S.R., A.K.W., and S.P.

## Supplementary Material

vdaa030_suppl_Supplementary_Information

## References

[1] Auf G, Jabouille A, Guérit S, et al. Inositol-requiring enzyme 1α is a key regulator of angiogenesis and invasion in malignant glioma. Proc Natl Acad Sci U S A. 2010;107(35):15553–15558. 20702765 10.1073/pnas.0914072107 PMC2932600

[2] Angara K, Borin TF, Arbab AS Vascular mimicry: a novel neovascularization mechanism driving anti-angiogenic therapy (AAT) resistance in glioblastoma. Transl Oncol. 2017;10(4):650–660. 28668763 10.1016/j.tranon.2017.04.007 PMC5496207

[3] Wang R, Chadalavada K, Wilshire J, et al. Glioblastoma stem-like cells give rise to tumour endothelium. Nature. 2010;468(7325):829–833. 21102433 10.1038/nature09624

[4] Hardee ME, Zagzag D Mechanisms of glioma-associated neovascularization. Am J Pathol. 2012;181(4):1126–1141. 22858156 10.1016/j.ajpath.2012.06.030 PMC3463636

[5] Dimberg A The glioblastoma vasculature as a target for cancer therapy. Biochem Soc Trans. 2014;42(6):1647–1652. 25399584 10.1042/BST20140278

[6] Dubois LG, Campanati L, Righy C, et al. Gliomas and the vascular fragility of the blood brain barrier. Front Cell Neurosci. 2014;8:418. 25565956 10.3389/fncel.2014.00418 PMC4264502

[7] Sarkaria JN, Hu LS, Parney IF, et al. Is the blood-brain barrier really disrupted in all glioblastomas? A critical assessment of existing clinical data. Neuro Oncol. 2018;20(2):184–191. 29016900 10.1093/neuonc/nox175 PMC5777482

[8] Junck L Bevacizumab antiangiogenic therapy for glioblastoma. Neurology. 2011;76(5):414–415. 21282587 10.1212/WNL.0b013e31820a0d7e

[9] Johnson DR, Leeper HE, Uhm JH Glioblastoma survival in the United States improved after food and drug administration approval of bevacizumab: a population-based analysis. Cancer. 2013;119(19):3489–3495. 23868553 10.1002/cncr.28259

[10] Brighi C, Puttick S, Rose S, Whittaker AK The potential for remodelling the tumour vasculature in glioblastoma. Adv Drug Deliv Rev. 2018;136–137:49–61. 10.1016/j.addr.2018.10.001 30308226

[11] Kim SS, Harford JB, Pirollo KF, Chang EH Effective treatment of glioblastoma requires crossing the blood-brain barrier and targeting tumors including cancer stem cells: the promise of nanomedicine. Biochem Biophys Res Commun. 2015;468(3):485–489. 26116770 10.1016/j.bbrc.2015.06.137 PMC4690805

[12] Rodriguez A, Tatter SB, Debinski W Neurosurgical techniques for disruption of the blood-brain barrier for glioblastoma treatment. Pharmaceutics. 2015;7(3):175–187. 26247958 10.3390/pharmaceutics7030175 PMC4588193

[13] Lamsam L, Johnson E, Connolly ID, Wintermark M, Hayden Gephart M A review of potential applications of MR-guided focused ultrasound for targeting brain tumor therapy. Neurosurg Focus. 2018;44(2):E10. 10.3171/2017.11.FOCUS17620 29385922

[14] Jolesz FA MRI-guided focused ultrasound surgery. Annu Rev Med. 2009;60:417–430. 19630579 10.1146/annurev.med.60.041707.170303 PMC4005559

[15] Lin Y-J, Chen K-T, Huang C-Y, Wei K-C Non-invasive focused ultrasound-based synergistic treatment of brain tumors. J Cancer Res Pract. 2016;3(3):63–68.

[16] Park J, Zhang Y, Vykhodtseva N, Jolesz FA, McDannold NJ The kinetics of blood brain barrier permeability and targeted doxorubicin delivery into brain induced by focused ultrasound. J Control Release. 2012;162(1):134–142. 22709590 10.1016/j.jconrel.2012.06.012 PMC3520430

[17] Park J, Aryal M, Vykhodtseva N, Zhang YZ, McDannold N Evaluation of permeability, doxorubicin delivery, and drug retention in a rat brain tumor model after ultrasound-induced blood-tumor barrier disruption. J Control Release. 2017;250:77–85. 27742444 10.1016/j.jconrel.2016.10.011 PMC5384106

[18] Wei KC, Chu PC, Wang HY, et al. Focused ultrasound-induced blood-brain barrier opening to enhance temozolomide delivery for glioblastoma treatment: a preclinical study. PLoS One. 2013;8(3):e58995. 23527068 10.1371/journal.pone.0058995 PMC3602591

[19] McDannold N, Zhang Y, Supko JG, et al. Acoustic feedback enables safe and reliable carboplatin delivery across the blood-brain barrier with a clinical focused ultrasound system and improves survival in a rat glioma model. Theranostics. 2019;9(21):6284–6299. 31534551 10.7150/thno.35892 PMC6735504

[20] Carpentier A, Canney M, Vignot A, et al. Clinical trial of blood-brain barrier disruption by pulsed ultrasound. Sci Transl Med. 2016;8(343):343re2. 10.1126/scitranslmed.aaf6086 27306666

[21] Kurz SC, Cabrera LP, Hastie D, et al. PD-1 inhibition has only limited clinical benefit in patients with recurrent high-grade glioma. Neurology. 2018;91(14):e1355–e1359. 30171077 10.1212/WNL.0000000000006283

[22] Filley AC, Henriquez M, Dey M Recurrent glioma clinical trial, CheckMate-143: the game is not over yet. Oncotarget. 2017;8(53): 91779–91794. 29207684 10.18632/oncotarget.21586 PMC5710964

[23] Reardon DA, Omuro A, Brandes AA, et al. OS10.3 Randomized phase 3 study evaluating the efficacy and safety of nivolumab vs bevacizumab in patients with recurrent glioblastoma: CheckMate 143. Neuro Oncol. 2017;19(Suppl 3):iii21.

[24] van den Bent M, Gan HK, Lassman AB, et al. Efficacy of depatuxizumab mafodotin (ABT-414) monotherapy in patients with EGFR-amplified, recurrent glioblastoma: results from a multi-center, international study. Cancer Chemother Pharmacol. 2017;80(6):1209–1217. 29075855 10.1007/s00280-017-3451-1 PMC5686264

[25] Kinoshita M, McDannold N, Jolesz FA, Hynynen K Targeted delivery of antibodies through the blood-brain barrier by MRI-guided focused ultrasound. Biochem Biophys Res Commun. 2006;340(4):1085–1090. 16403441 10.1016/j.bbrc.2005.12.112

[26] Raymond SB, Treat LH, Dewey JD, McDannold NJ, Hynynen K, Bacskai BJ Ultrasound enhanced delivery of molecular imaging and therapeutic agents in Alzheimer’s disease mouse models. PLoS One. 2008;3(5):e2175. 18478109 10.1371/journal.pone.0002175 PMC2364662

[27] Jordão JF, Ayala-Grosso CA, Markham K, et al. Antibodies targeted to the brain with image-guided focused ultrasound reduces amyloid-beta plaque load in the TgCRND8 mouse model of Alzheimer’s disease. PLoS One. 2010;5(5):e10549. 20485502 10.1371/journal.pone.0010549 PMC2868024

[28] Janowicz PW, Leinenga G, Götz J, Nisbet RM Ultrasound-mediated blood-brain barrier opening enhances delivery of therapeutically relevant formats of a tau-specific antibody. Sci Rep. 2019;9(1):9255. 31239479 10.1038/s41598-019-45577-2 PMC6592925

[29] Kinoshita M, McDannold N, Jolesz FA, Hynynen K Noninvasive localized delivery of Herceptin to the mouse brain by MRI-guided focused ultrasound-induced blood-brain barrier disruption. Proc Natl Acad Sci U S A. 2006;103(31):11719–11723. 16868082 10.1073/pnas.0604318103 PMC1544236

[30] Park EJ, Zhang YZ, Vykhodtseva N, McDannold N Ultrasound-mediated blood-brain/blood-tumor barrier disruption improves outcomes with trastuzumab in a breast cancer brain metastasis model. J Control Release. 2012;163(3):277–284. 23000189 10.1016/j.jconrel.2012.09.007 PMC3502612

[31] Chen PY, Hsieh HY, Huang CY, Lin CY, Wei KC, Liu HL Focused ultrasound-induced blood-brain barrier opening to enhance interleukin-12 delivery for brain tumor immunotherapy: a preclinical feasibility study. J Transl Med. 2015;13:93. 25784614 10.1186/s12967-015-0451-y PMC4369363

[32] Kobus T, Zervantonakis IK, Zhang Y, McDannold NJ Growth inhibition in a brain metastasis model by antibody delivery using focused ultrasound-mediated blood-brain barrier disruption. J Control Release. 2016;238:281–288. 27496633 10.1016/j.jconrel.2016.08.001 PMC5014601

[33] Liu HL, Hsu PH, Lin CY, et al. Focused ultrasound enhances central nervous system delivery of bevacizumab for malignant glioma treatment. Radiology. 2016;281(1):99–108. 27192459 10.1148/radiol.2016152444

[34] Treat LH, McDannold N, Vykhodtseva N, Zhang Y, Tam K, Hynynen K Targeted delivery of doxorubicin to the rat brain at therapeutic levels using MRI-guided focused ultrasound. Int J Cancer. 2007;121(4):901–907. 17437269 10.1002/ijc.22732

[35] Mei J, Cheng Y, Song Y, et al. Experimental study on targeted methotrexate delivery to the rabbit brain via magnetic resonance imaging-guided focused ultrasound. J Ultrasound Med. 2009;28(7):871–880. 19546329 10.7863/jum.2009.28.7.871

[36] Beccaria K, Canney M, Goldwirt L, et al. Ultrasound-induced opening of the blood-brain barrier to enhance temozolomide and irinotecan delivery: an experimental study in rabbits. J Neurosurg. 2016;124(6):1602–1610. 26566207 10.3171/2015.4.JNS142893

[37] Day BW, Stringer BW, Al-Ejeh F, et al. EphA3 maintains tumorigenicity and is a therapeutic target in glioblastoma multiforme. Cancer Cell. 2013;23(2):238–248. 23410976 10.1016/j.ccr.2013.01.007

[38] Stringer BW, Day BW, D’Souza RCJ, et al. A reference collection of patient-derived cell line and xenograft models of proneural, classical and mesenchymal glioblastoma. Sci Rep. 2019;9(1):4902. 30894629 10.1038/s41598-019-41277-z PMC6427001

[39] Puttick S, Stringer BW, Day BW, et al. EphA2 as a diagnostic imaging target in glioblastoma: a positron emission tomography/magnetic resonance imaging study. Mol Imaging. 2015;14:385–399. 26218510

[40] Zeglis BM, Lewis JS The bioconjugation and radiosynthesis of 89Zr-DFO-labeled antibodies. J Vis Exp. 2015;( 96):e52521. 10.3791/52521 PMC4354640 25741890

[41] dcm2niix. https://github.com/rordenlab/dcm2niix. Accessed September 20, 2019.

[42] Tournier JD, Smith R, Raffelt D, et al. MRtrix3: a fast, flexible and open software framework for medical image processing and visualisation. Neuroimage. 2019;202:116137. 31473352 10.1016/j.neuroimage.2019.116137

[43] Avants BB, Tustison NJ, Song G, Cook PA, Klein A, Gee JC A reproducible evaluation of ANTs similarity metric performance in brain image registration. Neuroimage. 2011;54(3): 2033–2044. 20851191 10.1016/j.neuroimage.2010.09.025 PMC3065962

[44] Yushkevich PA, Piven J, Hazlett HC, et al. User-guided 3D active contour segmentation of anatomical structures: significantly improved efficiency and reliability. Neuroimage. 2006;31(3): 1116–1128. 16545965 10.1016/j.neuroimage.2006.01.015

[45] Jenkinson M, Beckmann CF, Behrens TE, Woolrich MW, Smith SM FSL. Neuroimage. 2012;62(2):782–790. 21979382 10.1016/j.neuroimage.2011.09.015

[46] Tofts PS T1-weighted DCE imaging concepts: modelling, acquisition and analysis. Magnetom Flash. 2010;3(450):30–39.

[47] Arvin B, Neville LF, Barone FC, Feuerstein GZ The role of inflammation and cytokines in brain injury. Neurosci Biobehav Rev. 1996;20(3):445–452. 8880734 10.1016/0149-7634(95)00026-7

[48] O’Brien ER, Howarth C, Sibson NR The role of astrocytes in CNS tumors: pre-clinical models and novel imaging approaches. Front Cell Neurosci. 2013;7(Mar):40. 23596394 10.3389/fncel.2013.00040 PMC3627137

[49] Kovacs ZI, Kim S, Jikaria N, et al. Disrupting the blood-brain barrier by focused ultrasound induces sterile inflammation. Proc Natl Acad Sci U S A. 2017;114(1):E75–E84. 27994152 10.1073/pnas.1614777114 PMC5224365

[50] Sinharay S, Tu TW, Kovacs ZI, et al. In vivo imaging of sterile microglial activation in rat brain after disrupting the blood-brain barrier with pulsed focused ultrasound: [18F]DPA-714 PET study. J Neuroinflammation. 2019;16(1):155. 31345243 10.1186/s12974-019-1543-z PMC6657093

[51] da Fonseca AC, Matias D, Garcia C, et al. The impact of microglial activation on blood-brain barrier in brain diseases. Front Cell Neurosci. 2014;8(November):362. 25404894 10.3389/fncel.2014.00362 PMC4217497

[52] Silburt J, Lipsman N, Aubert I Disrupting the blood-brain barrier with focused ultrasound: perspectives on inflammation and regeneration. Proc Natl Acad Sci U S A. 2017;114(33):E6735–E6736. 28798067 10.1073/pnas.1710761114 PMC5565470

[53] Arvanitis CD, Askoxylakis V, Guo Y, et al. Mechanisms of enhanced drug delivery in brain metastases with focused ultrasound-induced blood-tumor barrier disruption. Proc Natl Acad Sci U S A. 2018;115(37):E8717–E8726. 30150398 10.1073/pnas.1807105115 PMC6140479

