# Supplementary material for: MR-guided focused ultrasound increases antibody delivery to nonenhancing high-grade glioma
Source: Neurooncol Adv. 2020 Mar 5;2(1):vdaa030. doi: 10.1093/noajnl/vdaa030 (PMC7212871; doi:10.1093/noajnl/vdaa030)
Supplement: vdaa030_suppl_Supplementary_Information [file vdaa030_suppl_supplementary_information.pdf]

Supplementary Figures

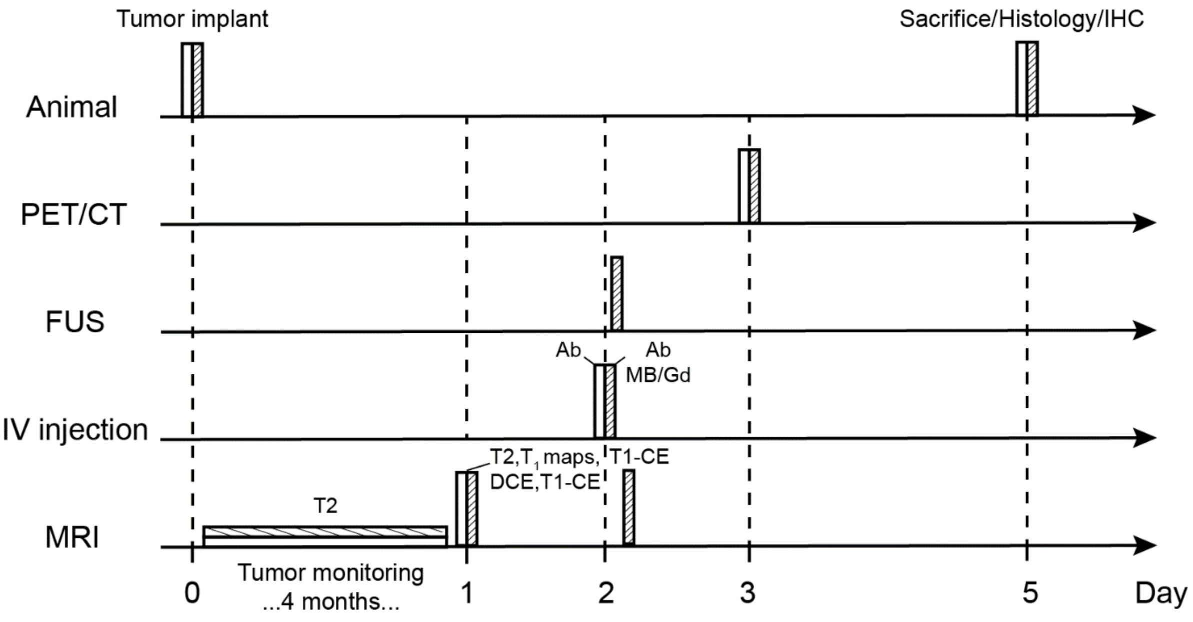

Fig. S1. *Experimental design*. Imaging and treatment time sequence used in this study. The control group is represented by empty bars, while the FUS group is represented by striped bars. After tumor implant the tumor growth was monitored with T2 images. Once the tumor reached 200 ± 100 mm^3 mice were enrolled in the experiment and T2, T_1 maps, DCE and T1-CE MR images were acquired for both groups on Day 1. The ^{89}Zr labelled EphA2 specific antibody was injected intravenously in all the mice on Day 2. For the FUS group, FUS was applied to the target volume during infusion of a solution of Definity™ microbubbles and gadolinium CA, and a T1-CE was acquired post-sonication. A PET-CT scan was acquired for both groups at 24 h. post-antibody injection (Day 3) and mice were sacrificed, and brains collected for histology and IHC on Day 5.

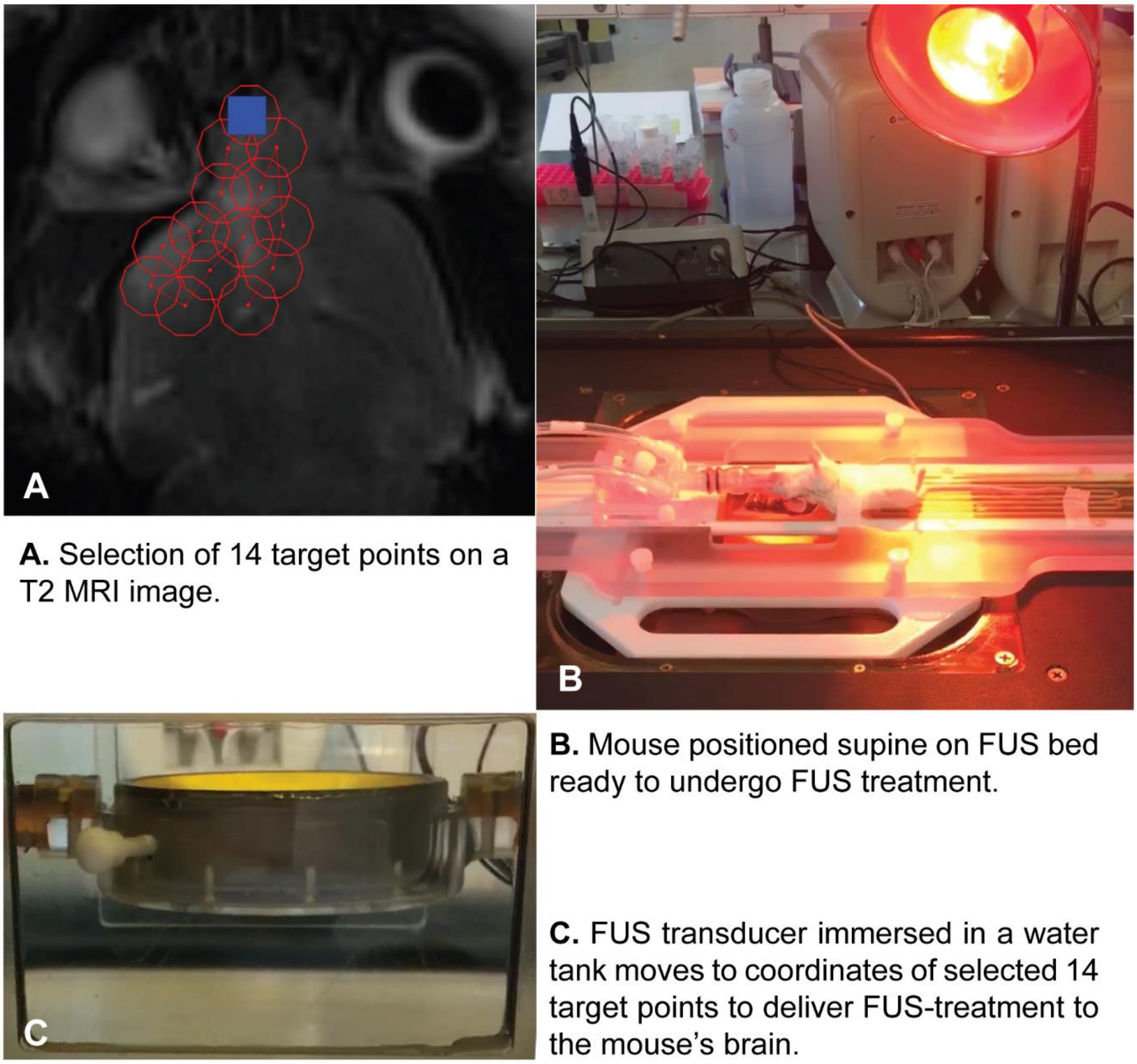

Fig. S2. *Experimental setup*. The diagrams illustrate **A)** coronal T2 image used to localize 14 target points in the entire tumor; **B)** the mouse anaesthetized and placed supine on the FUS bed kept warm with a heat lamp and undergoing sonication; **C)** focused ultrasound transducer submerged in a water tank directly below the mouse bed. During sonication the transducer moves to the coordinates of the 14 selected target points to deliver FUS pulses.

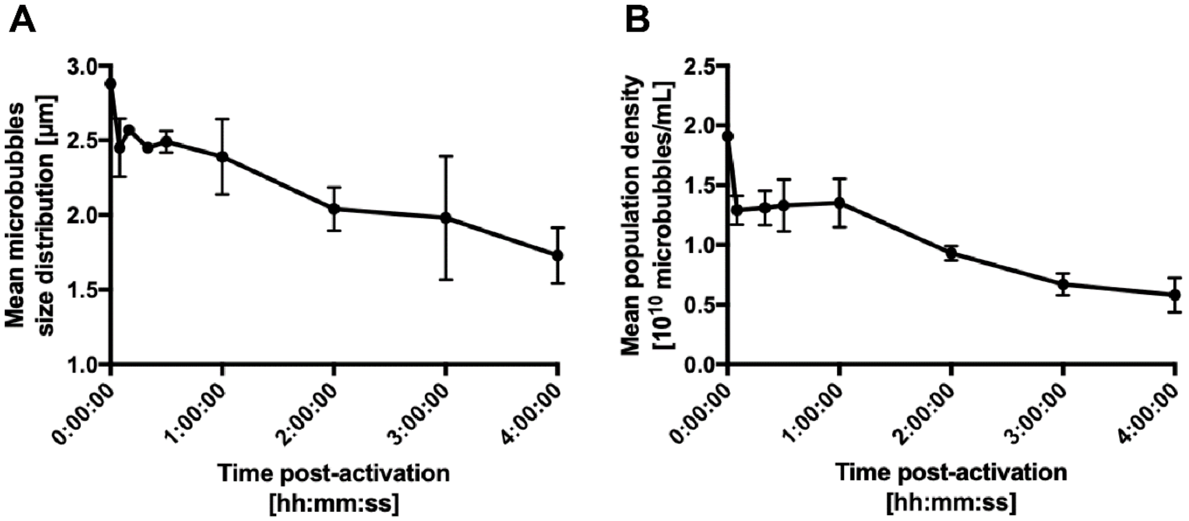

Fig. S3. *Definity™ microbubbles stability*. The plots illustrate **A)** the mean microbubbles size distribution and **B)** the mean microbubbles population density at 0, 5, 10 and 30 min, 1, 2, 3 and 4 h. post activation. Data were acquired with a coulter counter multisizer (Beckman).

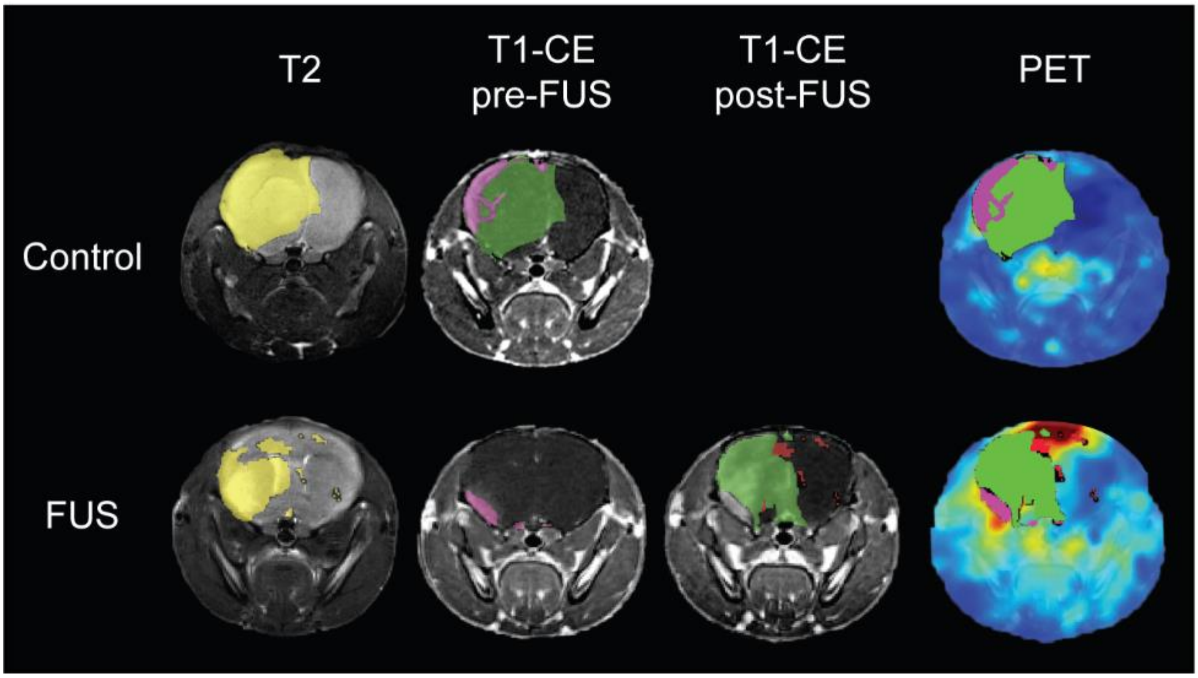

Fig. S4. *Overlays of masks of VOIs over MRI and PET images.* The figure illustrates (L-R) T2 images with overlayed tumor VOI mask (yellow), T1-CE pre-FUS with overlayed CE tumor VOIs mask (purple) for both mice and targeted non-CE tumor VOI mask (green) for the control mouse, T1-CE post-FUS with overlayed targeted non-CE tumor VOI mask (green) and non-CE tumor post-FUS VOI mask (red), and PET images with overlayed masks of CE tumor VOI (purple), targeted non-CE tumor VOI (green) and non CE-tumor post-FUS VOI (red). Note that for the control mouse the non-CE tumor post-FUS VOI is not highlighted as it corresponds to the targeted non-CE tumor VOI (green).

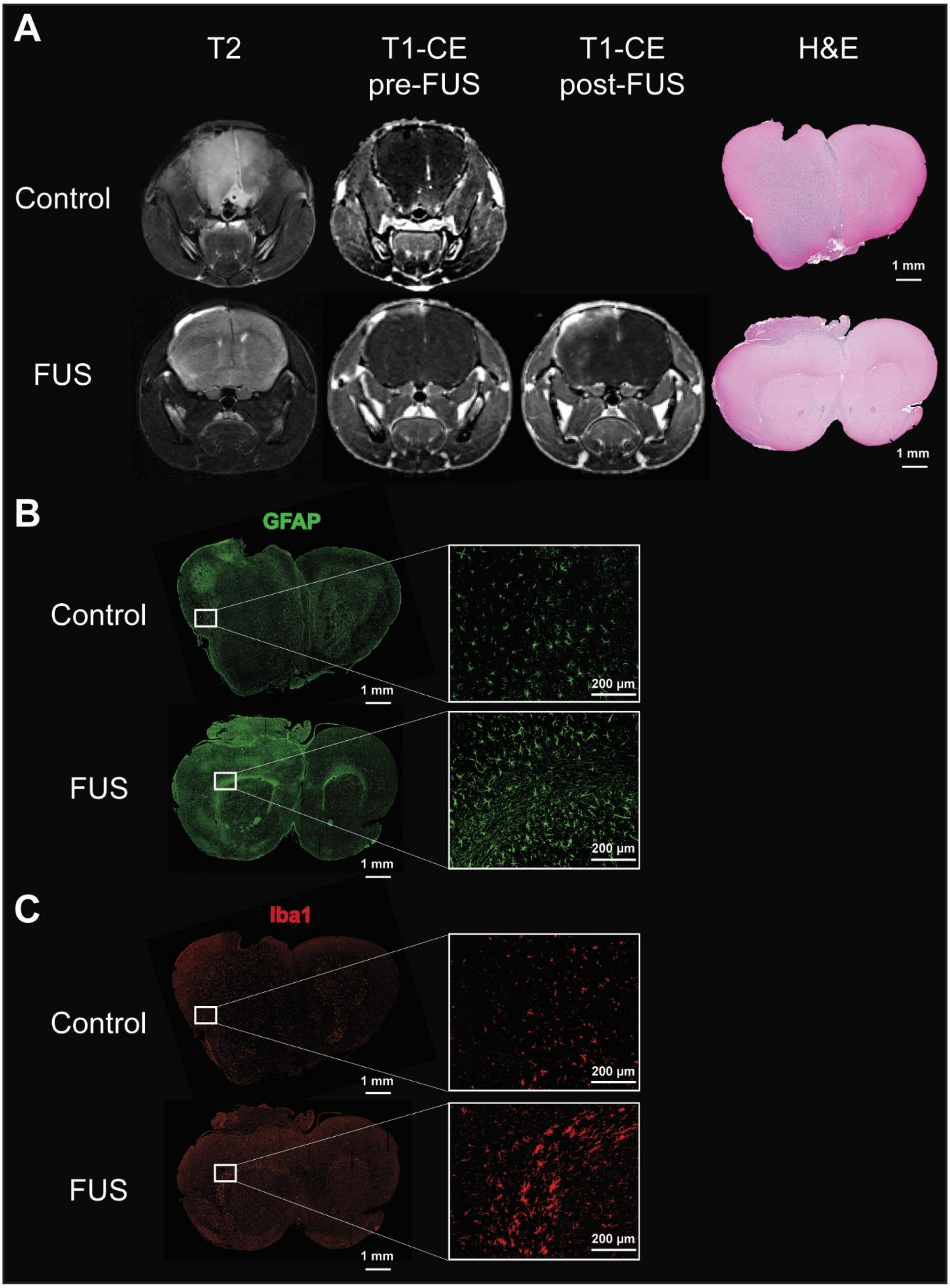

Fig. S5. *Another* example *tumor characteristics and FUS treatment effects for a control mouse and a FUS mouse.* **A)** T2 images, T1-CE pre-FUS, T1-CE post-FUS images and H&E stained brain sections; **B)** GFAP stained

sections; **C)** Iba1 stained sections. Higher-magnification (20X) images of GFAP and Iba1 stained sections are taken in regions of non-CE tumor in the control mouse, and in regions of FUS-treated tumor in the FUS mouse.
